# Extensive hybridization following a large escape of domesticated Atlantic salmon in the Northwest Atlantic

**DOI:** 10.1038/s42003-018-0112-9

**Published:** 2018-08-09

**Authors:** Brendan F. Wringe, Nicholas W. Jeffery, Ryan R. E. Stanley, Lorraine C. Hamilton, Eric C. Anderson, Ian A. Fleming, Carole Grant, J. Brian Dempson, Geoff Veinott, Steven J. Duffy, Ian R. Bradbury

**Affiliations:** 1 Science Branch, Fisheries and Oceans Canada, 80 East White Hills Road, St. John’s, Newfoundland, A1C 5X1 Canada; 2 Department of Ocean Sciences, Memorial University of Newfoundland, St. John’s, Newfoundland, A1C 5S7 Canada; 3 Faculty of Computer Science, Dalhousie University, Halifax, NS B3H 4R2 Canada; 4 Science Branch, Fisheries and Oceans Canada, Bedford Institute of Oceanography, Dartmouth, NS B2Y 4A2 Canada; 5 Aquatic Biotechnology Laboratory, Fisheries and Oceans Canada, Bedford Institute of Oceanography, Dartmouth, NS B2Y 4A2 Canada; 6 Fisheries Ecology Division, Southwest Fisheries Science Center, National Marine Fisheries Service, National Oceanic and Atmospheric Administration, Santa Cruz, CA 95060 USA

## Abstract

Domestication is rife with episodes of interbreeding between cultured and wild populations, potentially challenging adaptive variation in the wild. In Atlantic salmon, *Salmo salar*, the number of domesticated individuals far exceeds wild individuals, and escape events occur regularly, yet evidence of the magnitude and geographic scale of interbreeding resulting from individual escape events is lacking. We screened juvenile Atlantic salmon using 95 single nucleotide polymorphisms following a single, large aquaculture escape in the Northwest Atlantic and report the landscape-scale detection of hybrid and feral salmon (27.1%, 17/18 rivers). Hybrids were reproductively viable, and observed at higher frequency in smaller wild populations. Repeated annual sampling of this cohort revealed decreases in the presence of hybrid and feral offspring over time. These results link previous observations of escaped salmon in rivers with reports of population genetic change, and demonstrate the potential negative consequences of escapes from net-pen aquaculture on wild populations.

## Abstract (web summary)

Brendan Wringe et al. find evidence of extensive hybridization between wild and domesticated salmon following a large escape event in the Northwest Atlantic in 2013. Genetic screening of juvenile salmon shows that > 27% of fish in 17 of 18 rivers examined are hybrids or feral, demonstrating a significant impact of escaped individuals on local populations.

## Introduction

The process of domestication results in genetically-based phenotypic divergence from wild populations, through both intentional and unintentional selection^1–3^. Modern genomic data in both plant^4–6^ and animal systems^3,7,8^ have revealed that recurrent hybridization and gene flow between cultured and wild populations can occur, not only during the early stages of domestication^9^, but throughout the entire period of culture^10^. Repeated episodes of hybridization between cultured and wild populations can be detrimental for wild populations, resulting in the introduction of non-native alleles^6^, erosion of adaptive diversity in the wild^4,11^, and ultimately a loss-of-wild population viability^12,13^. The management and conservation of wild populations, confronted with domesticated conspecifics, requires the accurate quantification of potential genetic and ecological impacts to inform risk assessment and mitigation strategies.

The Atlantic salmon, *Salmo salar*, is of considerable socioeconomic value both in culture and in the wild. Domestication of Atlantic salmon was initiated in 1969 in Norway^14^, and separately in 1979 in Eastern Canada^15^. Despite this short period, the process of domestication has resulted in genetic differences between cultured and wild Atlantic salmon^16–18^ which are likely maladaptive, and lead to lower relative survival of cultured salmon in the wild^19^. Domesticated Atlantic salmon exhibit lower relative fitness and spawning success compared to wild Atlantic salmon^13,19–21^, and interbreeding can impart lasting, heritable, population-level reductions in fitness to wild populations^12^. Escapes from Atlantic salmon net-pen aquaculture are a regular occurrence^22^, and the number of escapees can equate to an appreciable fraction of, or exceed, wild census size^23,24^. As such, genetic changes in wild populations consistent with introgression from domesticated salmon have been detected in nearly all regions where salmon aquaculture and wild populations co-occur, including: Norway^25,26^, Ireland^27,28^, Northern Ireland^29,30^, and Canada^31^. Furthermore, methodological and theoretical improvements^32,33^ have allowed the degree of hybridization within a single river^29,30^ or the cumulative impact of introgression at large spatial scales (i.e., >100 populations in Norway^34,35^), to be resolved. Nonetheless, the unequivocal quantification of the magnitude and geographic scale of domestic-wild hybridization associated with single-escape events across a broad landscape of wild salmon populations has remained elusive.

**Fig. 1:**
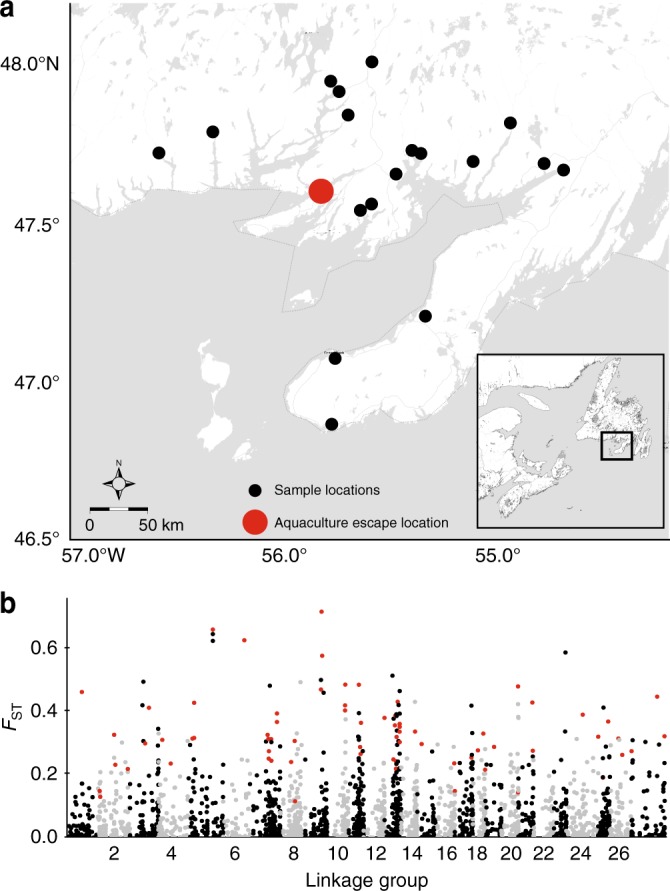
Geographic distribution of sampling relative to aquaculture escape event and genome-wide comparison of wild and domestic salmon. **a** Map of southern Newfoundland, location relative to eastern Canada shown in inset. Black dots represent rivers surveyed; the red dot denotes the location of the 2013 aquaculture escape event. **b** Manhattan plot illustrating the genome-wide genetic differentiation (*F*_ST_) between the wild and aquaculture baseline samples (Supplementary Table 7) used in the validation of the SNP panel accuracy. The red circles indicate the loci included in the 95 SNP collectively diagnostic panel. Linkage positions are from Brenna-Hansen et al.^64^

Here we quantify the presence and magnitude of hybridization between wild and escaped domestic individuals following an escape of ~20,000 sexually mature, domestic Atlantic salmon from a single aquaculture net-pen in southern Newfoundland, Canada. This event occured on September 18, 2013, just prior to the natural spawning period for salmon in this region (Fig. 1a). The southern Newfoundland region is analytically favorable for the detection of hybrids; because the domestic broodstock currently in use originates from a single-non-local source (Saint John River, New Brunswick, Canada), the magnitude of industry production in the region has been limited until recently, and finally estimates of the abundance of wild salmon throughout southern Newfoundland (~20,000 individuals) are approximately equal to the magnitude of the escape^22,36^. Juvenile salmon were collected from the region and screened using 95 single nucleotide polymorphisms (SNPs) to identify hybrids, hybrid classes, and feral individuals present following this escape event. Next, we evaluated factors influencing the distribution of hybrids, and the magnitude of hybridization. Finally, using repeated temporal sampling, we examined and compared relative changes in the abundance of various hybrid classes over time. We report widespread evidence of hybridization (27.1% and hybrids detected in 17/18 rivers) following this escape event. Hybrids were observed in higher frequency in smaller rivers, and repeated annual sampling revealed decreases in the presence of hybrid and feral offspring over time. These results demonstrate the potential genetic consequences of a single-escape event from net-pen aquaculture on wild Atlantic salmon populations.

## Results

### Hybrid identification and genomic-based screening

**Fig. 2:**
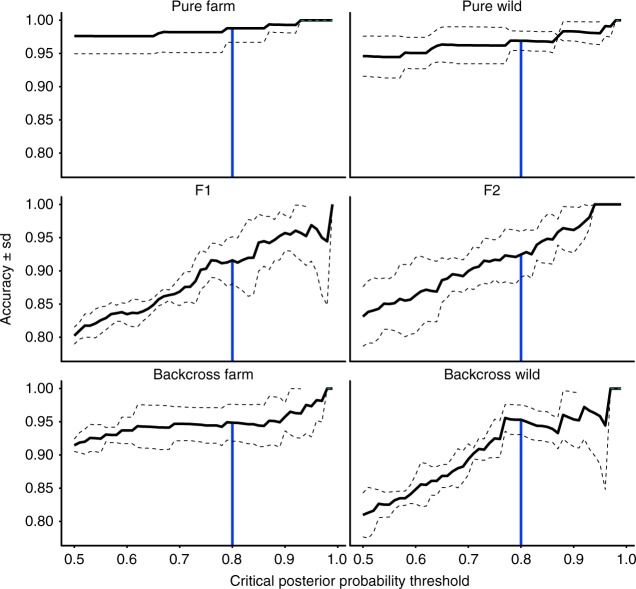
Accuracy of detection of each of the genotype frequency classes across a range of critical posterior probability thresholds for the 95 SNP panel used in this study. The black line represents the mean of three replicate analyses of each of three independently simulated datasets and the dotted lines are the standard deviation. The vertical blue line is meant to highlight the critical posterior probability of assignment threshold (>0.8) used in this study

In 2014, we collected 1704 young-of-the-year (YoY; i.e., fertilized the fall of the year of the escape, and hatched in the spring of the year of sampling) salmon from 18 rivers in the area adjacent to the escape event (Fig. 1; Table 1). Samples were again collected in 2015 (*n* = 836 of YoY and the 2014 cohort as 1+ juveniles; Table 1). All samples were screened using 95 genome-wide SNPs that were selected to maximize hybrid identification power and accuracy (Fig. 2; see Methods for further details as well as Supplementary Figures 1-3).

**Table 1:** Sample sizes of the juvenile Atlantic salmon screened for hybridization and introgression, the river from which they were collected, and the location of the river mouths

River name	Abbreviation	2104 YoY	2015 1+	2015 YoY	Lat (°N)	Long (°W)
Bottom Brook	BTB	32	33	0	47.765	56.322
Conne River	CNR	370	0	20	47.866	55.765
Dollard’s Brook	DLR	25	24	22	47.708	56.555
Northwest Brook	FBN	41	0	0	47.720	55.393
Garnish River	GAR	199	50	56	47.239	55.353
Grand Bank Brook	GBB	42	26	15	47.104	55.754
Grand LaPierre	GLP	118	76	14	47.674	54.781
Long Harbour River	LHR	137	94	49	47.780	54.948
Salmonier Brook	LMS	40	22	89	46.865	55.775
Little River	LTR	130	0	0	47.809	55.743
Mal Bay Brook	MAL	17	70	36	47.669	55.131
Northeast Brook	NEB	115	19	0	47.723	55.367
Old Bay Brook	OBB	18	0	0	47.563	55.593
Southeast Brook	SEB	31	19	0	47.920	55.750
Simm’s Brook	SMB	69	53	30	47.641	55.458
Taylor Bay Brook	TBB	120	0	0	47.543	55.637
Terrenceville Brook	TEB	120	0	0	47.671	54.711
Tailrace Brook	TRB	80	50	0	47.940	55.772

**Fig. 3:**
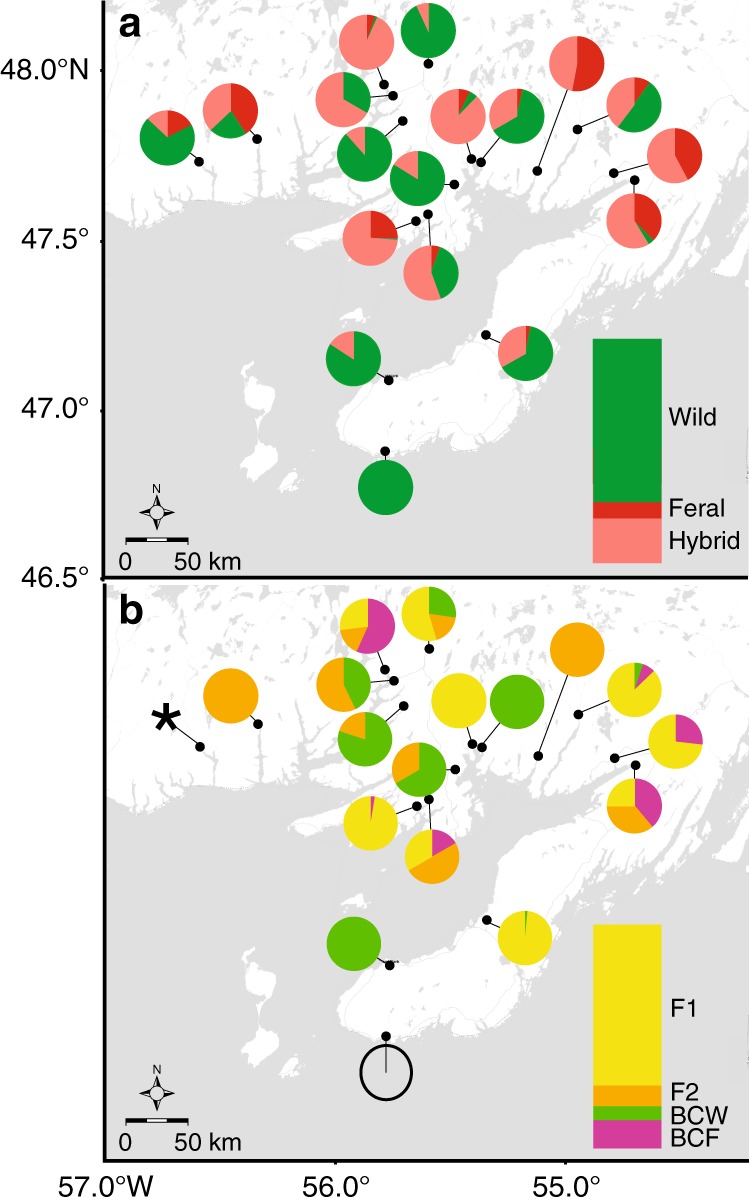
Distribution and extent of hybridization following a large escape event of domestic Atlantic salmon. **a** Geographic distribution of wild, feral, or hybrid young-of-the-year Atlantic salmon across sample locations in 2014. **b** River-specific proportions of hybrid young-of-the-year salmon partitioned by hybrid genotype class (i.e., F_1_, F_2_, backcross wild (BCW), and backcross farm (BCF)). The open circle indicates a sample in which no hybrids were found, the asterisk signifies a location where accurate assignment to hybrid class was not possible. Bar graphs represent the overall proportions of each class in the entire sampling range, taking into account the varying sizes of the sampled populations (i.e., weighting by the axial distance, the distance along a straight line along the longest axis of the river), and colors therein are used as the legend

Our panel of 95 highly informative genome-wide SNPs identified 27.1% of the sampled YoY in 2014 as being of aquaculture ancestry based on a posterior probability assignment >0.80 (i.e., any of feral, F_1_, F_2_, and backcrosses, Fig. 3a). Hybrids were detected in 17 of the 18 rivers sampled (Fig. 3a, b), and feral (i.e., offspring of two domestic salmon) offspring were detected in 13 rivers (Fig. 3a), revealing that the impacts of this escape event were substantial and region-wide. F_1_ hybrids were the most common hybrid class detected in 2014, but F_2_ and backcross individuals were also present (Fig. 3b). Observations of post-F_1_ hybrids (i.e., F_2_ and backcrosses) in 2014 YoY reveals that escape events had occurred prior to 2013, and that genetic introgression was occurring in some rivers. Observations of feral offspring indicative of successful reproduction among escapees has not been previously reported to our knowledge within the natural range of Atlantic salmon^18^. However, the potential for the establishment of feral populations remains unclear. Sibship reconstruction revealed multiple unique parents for the hybrid and feral individuals in each river, suggesting that the over representation of a few families did not skew the river-specific estimation of hybrid proportion (Supplementary Tables 1-3).

### Factors influencing hybridization

**Fig. 4:**
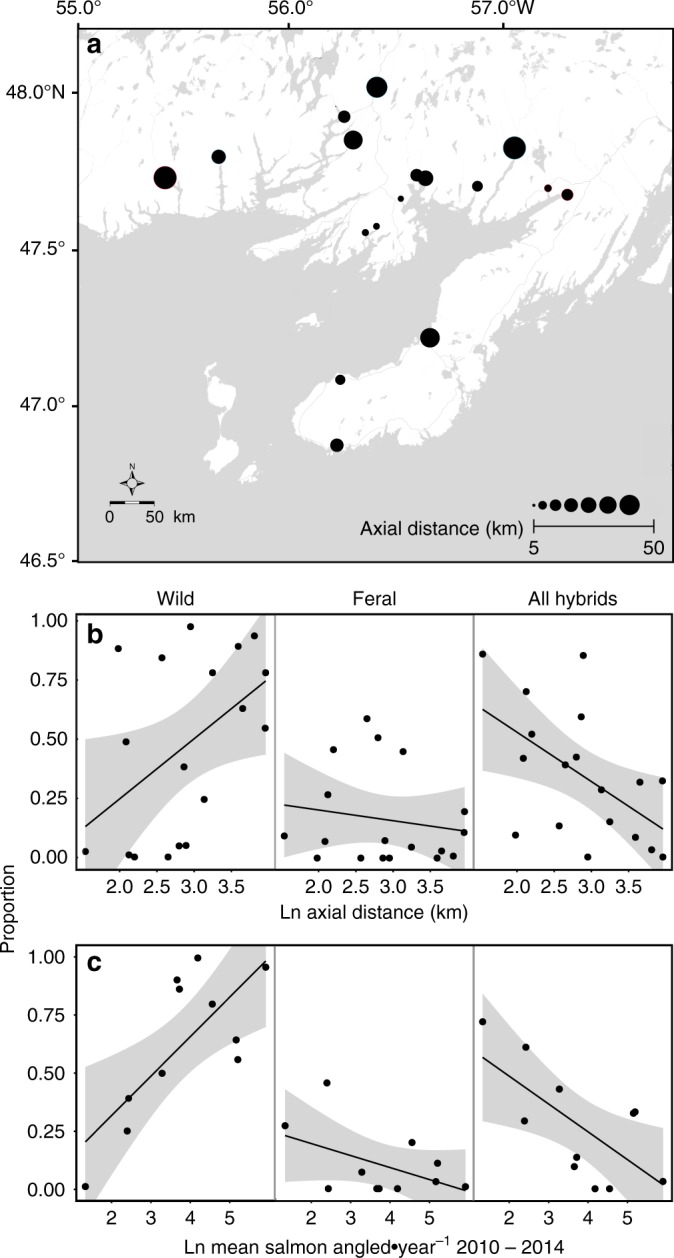
Association between wild population size and levels of hybridization. **a** River axial distance (i.e., the distance along a straight line along the longest axis of the river). **b** Relationship between river axial distance and the proportions of wild, feral, and all hybrid (i.e., sum of proportions of F_1_, F_2_, BC wild, and BC farm) young-of-the-year Atlantic salmon sampled in 2014. **c** Relationship between mean number of salmon angled (2010–2014) and the proportions of wild, feral, and hybrid young-of-the-year Atlantic salmon sampled in 2014. The gray shading is the 95% CI of the prediction of the linear models. See Supplementary Table 4 for model parameter estimates

Levels of hybridization detected in 2014 were significantly associated with wild population size (Fig. 4; Supplementary Table 4). This was evident in significant associations between levels of hybridization and two proxies for salmon population size: river axial distance (i.e., the length of a straight line along a river’s path), and average annual angling harvest (2010–2014), which correlate with salmon population size in this region (see Methods and Supplementary Figure 4). The proportion of hybrid YoY was negatively related to axial distance (Fig. 4b) and average annual angling (Fig. 4c); whereas, the opposite was true of the proportion of wild YoY (both *p* < 0.001, Supplementary Table 4). However, there was no statistical relationship between the proportion of feral YoY and either axial distance or average annual angling harvest (both *p* > 0.10; Supplementary Table 4). There was no evidence that distance between the location of the large escape event and river mouths influenced the proportion of wild, feral, or hybrid offspring detected in the year following the escape event (all *p* > 0.28; Supplementary Table 5).

### Temporal variation

**Fig. 5:**
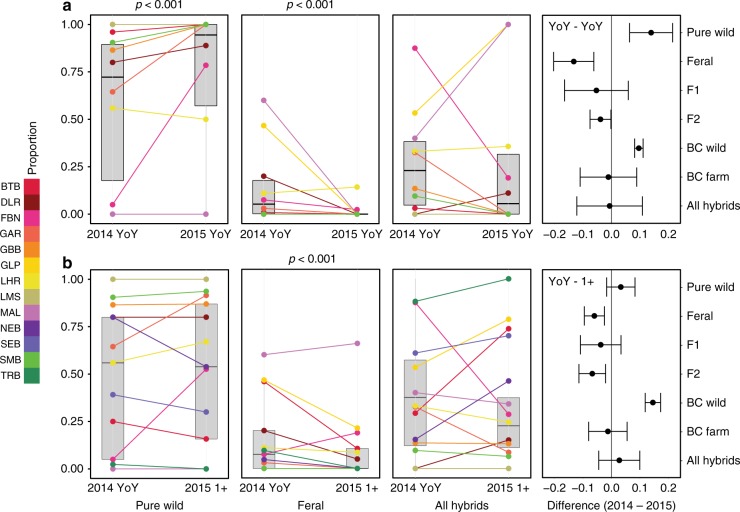
Temporal variation (2014–2015) in levels of hybridization. **a** River specific and overall trends in the proportion of wild, feral, and hybrid young-of-the-year Atlantic salmon between 2014 and 2015. Gray shaded boxplots illustrate the overall proportions across all rivers, midline represents the medians, the upper and lower bounds the interquartile ranges, and the whiskers extend to 1.5 times the interquartile range. Black dots represent the mean difference (±SE) between 2014 and 2015 in the proportion of each pure and hybrid class present. All hybrids is the sum of proportions of F_1_, F_2_, BC wild, and BC farm. **b** River specific and overall trends in the proportion of wild, feral, and hybrid young-of-the-year and one year old (1+) Atlantic salmon sampled in 2014 and 2015, respectively. Gray shaded boxplots illustrate the overall proportions across all rivers. The midline represents the medians, the upper and lower bounds the interquartile ranges, and the whiskers extend to 1.5 times the interquartile range. Black dots represent the mean difference (±SE) across rivers between 2014 and 2015 in the proportion of each pure and hybrid class present of young-of-the-year and 1-year-old individuals. All hybrids is the sum of proportions of F_1_, F_2_, BC wild, and BC farm. See Fig. 1 and Table 1 for location information, and sample sizes

To explore changes in the relative proportion of hybrids within the 2014 cohort over time, YoY and 1+juvenile salmon were sampled and analyzed in 2015 from across the region. In comparison to the YoY sampled in 2014, these 2015 YoY samples revealed an almost complete absence of feral individuals and declines in the prevalence of most hybrid classes. This is likely reflective of overall lower numbers of escapees in 2014, a year in which no escape events were reported (Fig. 5a). The decline in feral individuals was significant (*p* < 0.001), as was the consequent increase in the proportion wild (*p* < 0.001). However, whereas most hybrid classes were found to decrease, the change in the overall proportion of hybrids was offset by the increase in backcross wild individuals resulting in no significant difference between years (*p* = 0.56; Fig. 5a).

Potential offspring from the 2013 escape event (1+individuals in 2015), showed that the proportion classified as feral declined significantly after a single year of selection in the wild (*p* < 0.001; Fig. 5b). There was no significant difference in the proportion of wild individuals (*p* = 0.06) and while there were decreases in most hybrid groups, the increase in backcross wild individuals muted any consistent statistical trend between years and among rivers and hybrid classes (*p* = 0.20; Fig. 5b). This decrease in the prevalence of offspring with part or full domestic ancestry is consistent with the reductions in relative hybrid survivorship observed in experimental studies^13,21,37^ and expected selection against these individuals in the wild. Nonetheless, the continued presence of F_2_ and backcross individuals, as well as the observed increases in prevalence of wild backcross individuals indicates introgression is occurring.

## Discussion

We report unambiguous landscape-scale evidence of interbreeding between wild and escapee Atlantic salmon resulting from a single-escape event, and of particular note, the first documented instance of which we are aware of feral offspring within the native range of Atlantic salmon^18^. The combination of a highly informative panel of genome-wide SNPs with a large escape event of non-local domestic individuals into largely pristine wild populations allowed unprecedented resolution of the magnitude and geographic scale of hybridization following a single-escape event. Hybrid and feral offspring were widespread geographically, occurring at distances of up to100 km from the escape event, and accounted for ~27% of juvenile salmon surveyed. Moreover, the detection of F_2_ and backcross individuals, presumably resultant from previous escape events, strongly supports the continued survival and reproductive viability of some hybrids, as well as the potential for significant demographic and genetic change as reported elsewhere^18^.

Our results demonstrate a clear association between the size of wild populations and the degree of hybridization (Fig. 4) suggesting that smaller salmon populations are at greater risk of hybridization and introgression with escaped domestic individuals as noted in Norway^34,38^. This relationship is consistent with the dilution of domestic individuals in larger wild populations, as well the consequences of increased competition between wild and domestic individuals both on the spawning grounds and at juvenile stages^26,34^. Although, we lack actual estimates of wild population census size for many of the rivers included, the two correlates used here (river axial distance and annual angling harvest) are highly associated with population size on monitored rivers within the region (Supplementary Figure 5) and likely reflective of spatial trends in population census size.

Our results provide evidence consistent with declines in the proportion of offspring with domestic ancestry (e.g., hybrid, and feral) over time following the escape event. Comparison of the hybrid class composition of 1-year-old individuals sampled in 2015 relative to young-of-the-year sampled in 2014, revealed decreases in most hybrid classes, with only wild and wild backcrosses increasing in prevalence (Fig. 5b). Reduced wild-domestic hybrid survivorship for Atlantic salmon has previously been reported in experimental studies^13,21,37^, but, we believe this is the first documentation following a single-escape event in the wild (Fig. 5b). The observed loss of feral and hybrid individuals over time is consistent with expected selection against these individuals in the wild. Interestingly, hybrid class composition of young-of-the-year sampled in 2015 revealed an almost complete absence of feral individuals and declines in the prevalence of most hybrid classes. This is consistent with an absence of reported escape events in 2015 and a reduced influence of the 2013 escape event. Despite evidence of declines in the proportion of domestic offspring or hybrids over time, the continued presence of F_2_s and backcrosses is clear evidence of introgression and that significant genetic change is occurring in these wild populations^39^.

The identification and quantification of introgression and hybridization between domestic and wild Atlantic salmon is a critical first step toward understanding, predicting, and managing the genetic impacts of net-pen salmon aquaculture on wild populations. Our clear resolution of hybridization and introgression between escapee and wild Atlantic salmon in the Northwest Atlantic is the first to our knowledge, and is consistent with observations of genetic perturbation from aquaculture escapees^31^ both in the Canadian Maritimes and in Europe^14,34,35^. Our results link previous observations of escapes of domesticated Atlantic salmon with reports of population-level genetic changes^31,35^ and regional declines of Atlantic salmon populations^36^. Moreover, these results further demonstrate the potential consequences of escapes from net-pen aquaculture on wild Atlantic salmon populations.

## Methods

### Development of collectively diagnostic SNP panel

The collection of wild samples used for the development of our single nucleotide polymorphism (SNP) panel has been previously detailed in Bradbury et al.^40^. Briefly, juvenile Atlantic salmon (*n* = 260, 0+ to 3+ years of age), were collected via electrofishing during the summers of 2008–2010 (sample sizes are found in Supplementary Table 6; genetic differentiation between populations are described in Supplementary Table 7). All wild collections were conducted under the auspices of Fisheries and Oceans collection permits. Aquaculture samples (*n* = 156) were obtained from two cage sites located within the region shown in Fig. 1. No effort was made to screen for or remove potential sibs from these baseline groups^41^. These baseline individuals were first screened using a 5568 SNP-locus panel developed by the Centre for Integrative Genomics (CIGENE, Norway^42,43^) as per Bradbury et al.^44^. Locus calls were visually confirmed and loci were retained if call rates were >0.85 and with overall minor allele frequencies >0.01 or a minor allele frequency >0.05 in either population^44^. The loci retained after quality control filtering were ranked by Weir and Cockerham’s^45^ *F*_ST_ between the two pooled reference groups (wild and domestic salmon), and the 95 most informative loci for which suitable assays could be developed were incorporated into the custom Fluidigm EPI array (see below). Linkage disequilibrium was not considered explicitly, however, the final panel provided genome-wide coverage (Fig. 1).

For each candidate locus, sequences from identified targets were downloaded from GenBank (SNP database, www.ncbi.nlm.nih.gov) and submitted to D3 Assay Design application (www.d3.fluidigm.com) for SNP Type assay design (Fluidigm, San Francisco, CA, USA). Assays were tested on samples with known genotype and the selection criteria for inclusion in the final panel included: correct genotypes for known samples and positive controls (see below); genotypes being reproducible across multiple chip runs; the ranking of the target SNP in the prioritized list; and assays not requiring the STA (Specific Target Amplification) step. Positive controls consisted of normalized solutions of synthesized double stranded DNA (gBlocks (Integrated DNA Technologies, Coralville, IA, USA))^46^. SNP genotyping was performed using SNP type assays (Fluidigm) per the manufacturer’s protocols, without the STA (Specific Target Amplification) step, using 96.96 genotyping Integrated Fluidic Circuits (IFC) and read on an EP1 (Fluidigm) and analyzed using SNP Genotyping Analysis software (Fluidigm). Each 96-well plate extraction included 10 samples that were repeated on the plate (redundants) to detect processing errors (row or plate reversal) and ensure consistent clustering interpretation. The setup for each IFC also included positive controls (see above for details). To calculate the genotype error rate, 11.3% of the samples were reanalyzed from the original tissue where tissue samples were permitted. Based on Pompanon et al.^47^, the genotype error rate was calculated to be 0.01%.

### Hybrids

We used the R^48^ package hybriddetective^49^ to simulate pure wild, farmed, and hybrid populations to evaluate the power of this panel to identify hybrids and hybrid classes. Using hybriddetective we simulated multigenerational (viz. pure wild, pure farm, F_1_, F_2_, and backcrosses to wild and farm) hybrid datasets based on the genotypes of our wild and farmed baselines at the 95 SNPs in our panel. A random subset of 90% of the individuals from the wild and farmed baselines was first taken. A centered wild baseline was created by randomly sampling two alleles per locus from those of the randomly sampled subset without replacement. The same was done to create a centered farmed baseline. Centering was done following Karlsson et al.^33^ and has the effect of removing linkage and deviances from Hardy-Weinberg equilibrium that may have been present in a pooled sample of populations. Next, using the centered baselines, individuals in generation *t*+1 were created by randomly sampling without replacement one allele per locus from each of the parental populations (i.e., wild baseline subsample and farmed baseline subsample) at time *t*^49^. Three independently simulated datasets were each in turn analyzed three times in parallel using NewHybrids^32^ and the R package parallelnewhybrid^50^, with a burn-in of 50,000 followed by 100,000 sweeps. NEWHYBRIDS calculates the posterior probability that an individual belongs to each of, in our case, six hybrid classes^32^. The results of the analyses of these simulated datasets were used to determine the efficiency and accuracy^51^ of our 95 SNP panel.

To evaluate the efficacy of our panel, two metrics were considered: the panel’s accuracy and its efficiency. For both these measures, we use the definitions provided by Vähä and Primmer^51^. First, accuracy is the proportion of all individuals that were assigned to a hybrid class that truly belong in that hybrid class (i.e., number of individuals correctly assigned to a hybrid class divided by the total number of individuals assigned to that class), and is calculated independently for each hybrid class. Efficiency, is also calculated independently for each hybrid class, and measures the proportion of individuals that are known a priori to belong to a hybrid class that were assigned to that class (i.e., number of individuals correctly assigned to a hybrid class divided by total number of individuals known a priori to belong to a class). The accuracies and efficiencies calculated from the analyses of these simulated datasets across a range of posterior probability of assignment thresholds are shown in Fig. 2 and Supplementary Figures 1-3. From Fig. 2 (and also Supplementary Figure 3) it can be seen that the proportion of simulated individuals correctly assigned as either pure wild or feral are the highest across all posterior probability of assignment thresholds, while F_1_, F_2_, and backcross wild were comparatively lower. However, at all posterior probabilities of assignment shown, the accuracy for all hybrid classes was >80%, suggesting the potential impact of miss-assignments is low. Similarly, efficiencies (Supplementary Figures 1 and 2) were above 90% for posterior probability of assignments thresholds between 0.5 and 0.8 (used in this analysis), suggesting the majority of individuals were assigned. Taken together, the high accuracy indicates that of those individuals assigned to a given class the majority were assigned correctly (i.e., little false assignment bias), while the high efficiency suggests that most individuals were assigned. A posterior probability of assignment threshold of 0.8 for individual classification was chosen based on the simulations and calculation of efficiency and accuracy (Fig. 2, Supplementary Figures 1-3). Individuals which did not meet the 0.8 posterior probability threshold for any hybrid class were considered only for the assignment as wild, farmed, or hybrid, and excluded from analyses focusing on specific hybrid classes. Convergence of the MCMC chains in NewHybrids was also confirmed using *hybriddetective*^49^.

We evaluated both assignment to each of the six genotype frequency classes (Fig. 2 and Supplementary Figure 1), and pooled hybrid class identification (Supplementary Figures 2 and 3) separately, and accepted individual assignments to a class if their posterior probability of assignment to that class met, or exceeded a threshold of 0.8. We chose the threshold of 0.8, which is more conservative than what is typically used (e.g., 0.5^51,52^), because, we wanted to maximize the accuracy of assignments (Fig. 2 and Supplementary Figures 3).

### Sample collection and analysis

On 18 September 2013, 20,000 sexually mature Atlantic salmon weighing between 4.5 and 7 kg (10–15 lbs) escaped from an open cage culture facility in southern Newfoundland, Canada. A number of these escapees were subsequently detected and captured in nearby rivers by technicians working for Fisheries and Oceans Canada (DFO). Gross morphological examination, in addition to necropsies conducted by DFO employees, showed that the recovered salmon were sexually mature, and in spawning condition. In 2014, the year following the large escape event, young-of-the-year (YoY) salmon were collected by electrofishing stream and river habitats in the 18 rivers shown in Fig. 1. Sampling included both rivers with historical records of established salmon populations (Conne River, Little River, Garnish River) and smaller streams lacking prior information on the presence or status of Atlantic salmon populations. With the exception of a few monitored rivers, information on the status of the wild populations in these rivers is largely lacking^36^; what information does exist suggests recent declines in abundance.

Individuals were approximately age-binned based on an expected size–size age distribution from 200 K aged Newfoundland parr with a 97.5% accuracy in YoY identification. All YoYs captured were euthanized and stored whole in 95% ethanol for later DNA extraction. Sample sizes by year for each river are listed in Table 1. Sampling was repeated in 2015 using the same methodology, with the exception that both YoY and 1+individuals were retained. The 1+individuals collected in 2015 belong to the same cohort of fish that were spawned following the escape event in 2013, and collected as YoY in 2014. Conversely, the YoY collected in the 2015 sampling were spawned in 2014, a year in which no escape events were reported in Newfoundland, and are thus expected to be reflective of the background rates of hybridization and introgression.

DNA was isolated from tissue samples using QIAamp 96 DNA QIAcube HT Kit (Qiagen, Toronto, ON, Canada) on a QIACube HT (Qiagen) per the manufacturer’s protocol with some modifications. Tissue samples were manually disrupted using a Tissue Lyser II (Qiagen) mixing 2 × 10 s at 20 s^−1^. DNA was eluted twice in 100 µL buffer AE (Qiagen) pre-heated to 70 °C. DNA extracts were quantified using Quant-iT PicoGreen dsDNA Assay Kit (Thermo Fisher Scientific, Waltham, MA, USA) and read on a FLUOStar OPTIMA fluorescence plate reader (BMG Labtech, Ortenberg, Germany). All individuals were screened using the custom Fluidigm SNP panel and NEWHYBRIDS was used to quantify the proportion of individuals from different genotype frequency classes present in a river sample^32^. Samples from each river, and each year, were run independently. Prior information on allele frequencies of baseline farm and wild salmon were also provided to NEWHYBRIDS during analyses by including simulated pure farm and pure wild individuals (i.e., the same individuals used in the testing of the accuracy and efficiency of the panel described above). The known class (i.e., pure wild and pure farm) were indicated to NEWHBYRIDS, as well as the fact that they were not to be included as part of the mixture^53^. Like in the determination of the efficacy of our panel described above, NEWHBYRIDS was run with a burn-in of 50,000 followed by 100,000 sweeps, which was found to be sufficient to ensure convergence during the panel testing. Proportions assigned to the various hybrid classes are shown in Supplementary Tables 8-10.

COLONY^54^ was used to simultaneously infer the parentage and sibships of the YoY sampled in 2014, and the YoY and 1+individuals sampled in 2015. Each river, sampling year, and year class was analyzed separately in COLONY and parents were assigned an ancestry (wild, farm, or F_1_) based on the hybrid class in question (i.e., if an individual was feral, both parents must be farmed, if an individual was an F1, one parent must be farmed and the other wild, if an individual is an F2, both parents must themselves have been F1s, etc.). For each river, sampling year, and year class, locus-specific allelic dropout rates were estimated using the “missing” function in PLINK^55,56^, and these were provided to COLONY. Allele frequencies were estimated by COLONY from the data provided. In running COLONY, because all samples were wild caught, no information about numbers of candidate males or females provided. Both sexes were assumed to be polygynous, and “long” runs with “VeryHigh” precision were used. Because, we were not attempting to assign parentage, merely estimate the number of families present in each sample, and show that the proportions of hybrid classes detected was not the result of over representation of one, or a few families, the full-sib grouping for each individual with the highest probability was accepted. It should also be noted that because no parental genotypes were provided to COLONY, it was unable to meaningfully assign sexes to parents. Therefore, the total number of parents are presented.

### Statistical analyses

All statistical analyses were conducted in R version 3.4^48^. The proportion of wild, feral, and hybrid at each location was explored for associations with wild population size; in this case two proxies were used (axial river distance and average annual harvest). For the Newfoundland region, wild population size^57^ is associated with river axial distance^58^ (the distance along a straight line along the longest axis of the river; linear model, *R*^2^ = 0.6944, *F*_1,8_ = 18.18, *p* < 0.01; Supplementary Figure 4) and as such, axial distance was used as a proxy for population size. We also used average annual harvest (2010–2014) as a proxy of population size; because, the two were related (linear model, *F*_1,8_ = 40.47, *R*^2^ = 0.835, *p* < 0.001). Harvest statistics are collected annually by Fisheries and Oceans Canada^59^, and counts of population size and estimates of annual harvest were available for 10 rivers (Supplementary Table 11).

Exponential models for effect of distance from the escape event were used because straying of farmed salmon generally follows a negative exponential distribution^60^. The relationship between the proportion feral, wild, and hybrids detected in each river and the distance between the river mouths and the site of the escape were tested and fit using the R function *nls*. No significant relationships were found for distance from the escape event (all *p* > 0.28; Supplementary Table 5), so this factor was not considered further. The impact of the relative size of the native salmon populations in respective rivers on proportions was tested using linear models with the R function *lm*. The proportion wild, the proportion feral, and the proportion hybrid were tested separately as a function of axial distance, and then average annual angling harvest between 2010 and 2014.

We tested for differences in proportion of wild, feral, or hybrid individuals between years within the same cohort (i.e., the YoY collected in 2014 and the 1+collected in 2015), and between years with and without reported large escape events (i.e., YoY collected in 2014 and YoY collected in 2015) using binomial mixed-effects models with river as the random effect using the R function *glmer*^61^. The proportions of wild, feral, and hybrid were tested with separate models, and *p*-values were adjusted using the false discovery rate^62^.

### Data availability

Genotype, river characteristic, salmon and angling count data for this study are available in the Dryad Digital repository^63^ at: 10.5061/dryad.3k888n7

## Electronic supplementary material

Supplementary Information

Description of Additional Supplementary Information

Supplementary Data 1
