# Supplementary material for: Extensive hybridization following a large escape of domesticated Atlantic salmon in the Northwest Atlantic
Source: Commun Biol. 2018 Aug 9;1:108. doi: 10.1038/s42003-018-0112-9 (PMC6123692; doi:10.1038/s42003-018-0112-9)
Supplement: Supplementary file 1 — Supplementary Information [file 42003_2018_112_MOESM1_ESM.pdf]

Supplementary Table 1. Numbers of young-of-the-year Atlantic salmon sampled in 2014, and the number of unique families and numbers of parents determined by COLONY for each river. Note: the sum of the number of wild, farmed and F1 parents may not necessarily equal the total number of parents. While COLONY was able to identify all unique parents, unambiguous assignment to hybrid class was not possible for every juvenile salmon sampled, and the hybrid class of the juveniles were used to infer the origin of their parents (refer to materials and methods for further clarification).

River Name	Sample Size	Number of Families	Total Parents	Number Wild Parents	Number Farm Parents	Number F1 Parents
Bottom Brook	32	24	26	7	4	7
Conne River	370	274	268	251	2	2
Dollard's Brook	25	23	26	19	4	0
Northwest Brook	41	20	21	13	8	0
Garnish River	199	156	146	134	11	1
Grand Bank Brook	42	39	40	36	0	2
Grand LaPierre	118	51	39	9	23	5
Long Harbour River	137	118	98	66	20	2
Salmonier Brook	40	32	34	34	0	0
Little River	130	122	115	105	0	3
Mal Bay Brook	17	14	20	0	9	9
Northeast Brook	115	97	86	62	3	8
Old Bay Brook	18	16	21	11	3	5
Southeast Brook	31	24	28	14	0	11
Simm's Brook	69	50	46	41	0	5
Taylor Bay Brook	120	90	78	34	37	1
Terrenceville Brook	120	93	81	6	28	28

Tailrace Brook	80	66	58	4	12	22

Supplementary Table 2. Numbers of young-of-the-year Atlantic salmon sampled in 2015, and the number of unique families and numbers of parents determined by COLONY for each river. Note: the sum of the number of wild, farmed and F1 parents may not necessarily equal the total number of parents. While COLONY was able to identify all unique parents, unambiguous assignment to hybrid class was not possible for every juvenile salmon sampled, and the hybrid class of the juveniles were used to infer the origin of their parents (refer to materials and methods for further clarification).

River Name	Sample Size	Number of Families	Total Parents	Number Wild Parents	Number Farm Parents	Number F1 Parents
Bottom Brook	-	-	-	-	-	-
Conne River	20	20	22	22	0	0
Dollard's Brook	22	22	27	21	0	1
Northwest Brook	-	-	-	-	-	-
Garnish River	56	56	40	39	0	0
Grand Bank Brook	15	15	20	20	0	0
Grand LaPierre	14	7	9	5	4	0
Long Harbour River	49	39	46	38	7	1
Salmonier Brook	89	78	63	63	0	0
Little River	-	-	-	-	-	-
Mal Bay Brook	36	17	19	15	3	0
Northeast Brook	-	-	-	-	-	-
Old Bay Brook	-	-	-	-	-	-
Southeast Brook	-	-	-	-	-	-
Simm's Brook	20	19	21	21	0	0
Taylor Bay Brook	-	-	-	-	-	-

Terrenceville Brook	-	-	-	-	-	-
Tailrace Brook	-	-	-	-	-	-

Supplementary Table 3. Numbers of 1+ Atlantic salmon sampled in 2015, and the number of unique families and numbers of parents determined by COLONY for each river. Note: the sum of the number of wild, farmed and F1 parents may not necessarily equal the total number of parents. While COLONY was able to identify all unique parents, unambiguous assignment to hybrid class was not possible for every juvenile salmon sampled, and the hybrid class of the juveniles were used to infer the origin of their parents (refer to materials and methods for further clarification).

River Name	Sample Size	Number of Families	Total Parents	Number Wild Parents	Number Farm Parents	Number F1 Parents
Bottom Brook	33	28	30	10	2	7
Conne River	-	-	-	-	-	-
Dollard's Brook	24	24	31	24	2	1
Northwest Brook	-	-	-	-	-	-
Garnish River	50	44	49	47	2	0
Grand Bank Brook	26	25	34	30	1	0
Grand LaPierre	76	41	37	5	19	8
Long Harbour River	94	90	81	58	9	3
Salmonier Brook	22	11	21	21	0	0
Little River	-	-	-	-	-	-
Mal Bay Brook	70	53	43	8	26	5
Northeast Brook	19	19	24	15	2	2
Old Bay Brook	-	-	-	-	-	-
Southeast Brook	19	19	31	12	6	0
Simm's Brook	53	45	44	38	1	2
Taylor Bay Brook	-	-	-	-	-	-
Terrenceville Brook	-	-	-	-	-	-
Tailrace Brook	50	45	42	10	2	16

Supplementary Table 4. Linear model outputs of the impact of relative wild salmon population size on the proportions of wild, feral and all hybrid young-of-the-year salmon collected from rivers in 2014. F-stat is the F-statistic and D.F. are the degrees of freedom

Independent Variable	Dependent Variable	Multiple R^2	Slope (Std. Err.)	Intercept (Std. Err.)	F-stat; D.F.	p-value
Ln Angling	Proportion Wild	0.5558	0.1712 (0.0510)	-0.0256 (0.2055)	11.26; 1,9	< 0.01
Ln Angling	Proportion Feral	0.2307	-0.0516 (0.0314)	0.3011 (0.1266)	2.70; 1,9	0.13
Ln Angling	Proportion Hybrid	0.4588	-0.1200 (0.0433)	0.7245 (0.1744)	7.63; 1,9	< 0.05
Ln Axial Distance	Proportion Wild	0.2355	0.2604 (0.1173)	-0.2680 (0.3489)	4.93; 1,16	< 0.05
Ln Axial Distance	Proportion Feral	0.0267	-0.0464 (0.0700)	0.2981 (0.2082)	0.44; 1, 16	0.51
Ln Axial Distance	Proportion Hybrid	0.2973	-0.2141 (0.0823)	0.9610 (0.2447)	6.77; 1,16	< 0.05

Supplementary Table 5. Negative exponential model outputs of the relationship between the distance from the 2013 escape event and river mouths on the proportions of wild, feral and all hybrid young-of-the-year salmon collected from rivers in 2014. D.F. are the degrees of freedom

Independent Variable	Dependent Variable	R^2	Slope (Std. Err.)	Intercept (Std. Err.)	T value; D.F.	p-value
Distance to Escape Event (km)	Proportion Wild	0.0576	27.42 (23.93)	-1.10 (0.43)	1.10; 16	0.28
Distance to Escape Event (km)	Proportion Feral	0.1247	-80.03 (81.33)	-0.91 (0.77)	-0.98; 16	0.34
Distance to Escape Event (km)	Proportion Hybrid	0.0047	-10.40 (31.79)	-0.92 (0.43)	-0.33; 16	0.74

Supplementary Table 6. Sample sizes within the populations that made up the wild and farmed baselines. Whether the population is wild or farmed is noted, and for wild populations the location of the river is given.

River Name	Abbreviatio n	Sample Size	Wild/Farmed	Latitude (°N)	Longitude (°W)
Bay Du Nord River	BDN	52	Wild	47.71232	55.41702
Conne River	CNR	44	Wild	47.866	55.765
Dollard's Brook	DLR	30	Wild	47.738	56.586
Garnish River	CNR	30	Wild	47.228	55.347
Grey River	GRR	17	Wild	47.692	57.004
Long Harbour River	LHR	38	Wild	47.780	54.948
LaPoile River	LPR	20	Wild	47.79996	58.32075
Old Bay Brook	OBB	29	Wild	47.578	55.590
Domestic Sample 1	DS1	117	Farmed		
Domestic Sample 2	DS2	39	Farmed		

Supplementary Table 7. Genetic differentiation between baseline samples of Atlantic salmon from southern Newfoundland using 95 SNPs. F_{ST} above diagonal, p-value below diagonal. DS1 and DS2 represent samples of aquaculture salmon.

	DS1	BDN	CAN	DLR	GAR	GRR	LHR	LPR	OBB	DS2
DS1	--	0.221	0.331	0.222	0.298	0.231	0.299	0.253	0.247	0.127
BDN	0.001	--	0.061	0.047	0.077	0.041	0.036	0.058	0.044	0.266
CAN	0.001	0.001	--	0.105	0.159	0.115	0.063	0.099	0.117	0.358
DLR	0.001	0.001	0.001	--	0.068	0.004	0.055	0.109	0.033	0.291
GAR	0.001	0.001	0.001	0.001	--	0.061	0.085	0.149	0.061	0.354
GRR	0.001	0.001	0.001	0.183	0.001	--	0.059	0.125	0.030	0.321
LHR	0.001	0.001	0.001	0.001	0.001	0.001	--	0.106	0.054	0.345
LPR	0.001	0.001	0.001	0.001	0.001	0.001	0.001	--	0.114	0.234
OBB	0.001	0.001	0.001	0.001	0.001	0.001	0.001	0.001	--	0.310
DS2	0.001	0.001	0.001	0.001	0.001	0.001	0.001	0.001	0.001	--

Supplementary Table 8. Proportions of individuals assigned to hybrid classes at a posterior probability of assignment of 0.8 for YoY individuals captured in 2014.

River	Feral	Wild	F1	F2	BC Wild	BC Farm	Unassigned
BTB	0.344	0.188	0.000	0.219	0.000	0.000	0.250
CNR	0.008	0.908	0.016	0.005	0.000	0.008	0.054
DLR	0.160	0.640	0.000	0.000	0.000	0.000	0.200
FBN	0.073	0.049	0.854	0.000	0.000	0.000	0.024
GAR	0.030	0.638	0.317	0.000	0.000	0.005	0.010
GBB	0.000	0.762	0.000	0.000	0.000	0.119	0.119
GLP	0.415	0.000	0.347	0.000	0.127	0.000	0.110
LHR	0.095	0.482	0.248	0.000	0.022	0.015	0.139
LMS	0.000	1.000	0.000	0.000	0.000	0.000	0.000
LTR	0.000	0.823	0.000	0.015	0.000	0.062	0.100
MAL	0.529	0.000	0.000	0.353	0.000	0.000	0.118
NEB	0.035	0.591	0.000	0.000	0.000	0.113	0.261
OBB	0.056	0.389	0.111	0.167	0.056	0.000	0.222
SEB	0.000	0.323	0.000	0.258	0.000	0.194	0.258
SMB	0.000	0.826	0.000	0.029	0.000	0.058	0.087
TBB	0.250	0.008	0.642	0.000	0.017	0.000	0.083
TEB	0.358	0.033	0.075	0.108	0.117	0.000	0.308
TRB	0.050	0.013	0.125	0.075	0.263	0.000	0.475

Supplementary Table 9. Proportions of individuals assigned to hybrid classes at a posterior probability of assignment of 0.8 for YoY captured in 2015.

River	Feral	Wild	F1	F2	BC Wild	BC Farm	Unassigned
BTB	0.061	0.091	0.030	0.000	0.000	0.394	0.424
DLR	0.042	0.667	0.000	0.000	0.000	0.125	0.167
FBN	0.000	0.091	0.364	0.000	0.000	0.000	0.545
GAR	0.000	0.860	0.080	0.000	0.000	0.000	0.060
GBB	0.000	0.769	0.115	0.000	0.000	0.000	0.115
GLP	0.171	0.000	0.513	0.000	0.118	0.000	0.197
LHR	0.074	0.585	0.181	0.000	0.021	0.011	0.128
LMS	0.000	1.000	0.000	0.000	0.000	0.000	0.000
MAL	0.443	0.000	0.143	0.000	0.057	0.029	0.329
NEB	0.000	0.368	0.105	0.000	0.000	0.211	0.316
SEB	0.000	0.158	0.000	0.000	0.000	0.368	0.474
SMB	0.000	0.830	0.000	0.000	0.019	0.038	0.113
TRB	0.000	0.000	0.000	0.220	0.120	0.340	0.320

Supplementary Table 10. Proportions of individuals assigned to hybrid classes at a posterior probability of assignment of 0.8 for 1+ individuals captured in 2015

River	Feral	Wild	F1	F2	BC Wild	BC Farm	Unassigned
BTB	0.000	0.333	0.000	0.000	0.000	0.667	0.000
DLR	0.000	0.727	0.000	0.000	0.000	0.091	0.182
FBN							
GAR	0.000	0.929	0.000	0.000	0.000	0.000	0.071
GBB	0.000	1.000	0.000	0.000	0.000	0.000	0.000
GLP	0.000	0.000	0.786	0.000	0.000	0.000	0.214
LAM	0.000	1.000	0.000	0.000	0.000	0.000	0.000
LHR	0.122	0.429	0.265	0.000	0.041	0.000	0.143
MAL	0.000	0.000	0.000	0.028	0.000	0.778	0.194
NEB	0.000	0.000	0.000	0.000	0.000	1.000	0.000
SEB							
SMB	0.000	0.950	0.000	0.000	0.000	0.000	0.050
TRB	0.000	0.000	0.000	0.444	0.000	0.000	0.556

Supplementary Table 11. Identities and locations of the 10 rivers used in the calculation of the relationship between river size (i.e. axial distance; the distance along a straight line along the longest axis of the river) and Atlantic salmon population size. The average number of salmon harvested per year (Avg. Harvested/Yr) and the average number of salmon detected at Fisheries and Oceans Canada counting fences (Avg. Count/Yr) during the period 2009-2014 were used as proxies of population size. Data for axial distance is from Porter, et al. [1], for counts is from DFO [2] and harvest statistics is from DFO [3].

River	Avg. Harvested/Yr	Avg. Count/Year	Axial Distance	Latitude (°N)	Longitude (°W)
Campbellton River	559.0	4633.4	40.2	49.28	54.92
Conne River	370.8	1919.4	45.2	47.91	55.70
Exploits River	9362.2	36544.6	237.2	49.03	55.41
Gander River	3952.2	25733.3	156.7	49.26	54.49
Harry's River	1010.8	3319.4	58.6	48.51	58.42
Middle Brook	612.6	3105.2	41.8	48.24	58.83
Northwest River	45.8	1181.0	60.0	55.393	47.72
Rocky River	3.0	578.4	37.0	47.22	53.57
Terra Nova River	710.4	4492.2	105.4	48.67	54.00
Torrent River	730.8	4562.0	38.6	50.61	57.15

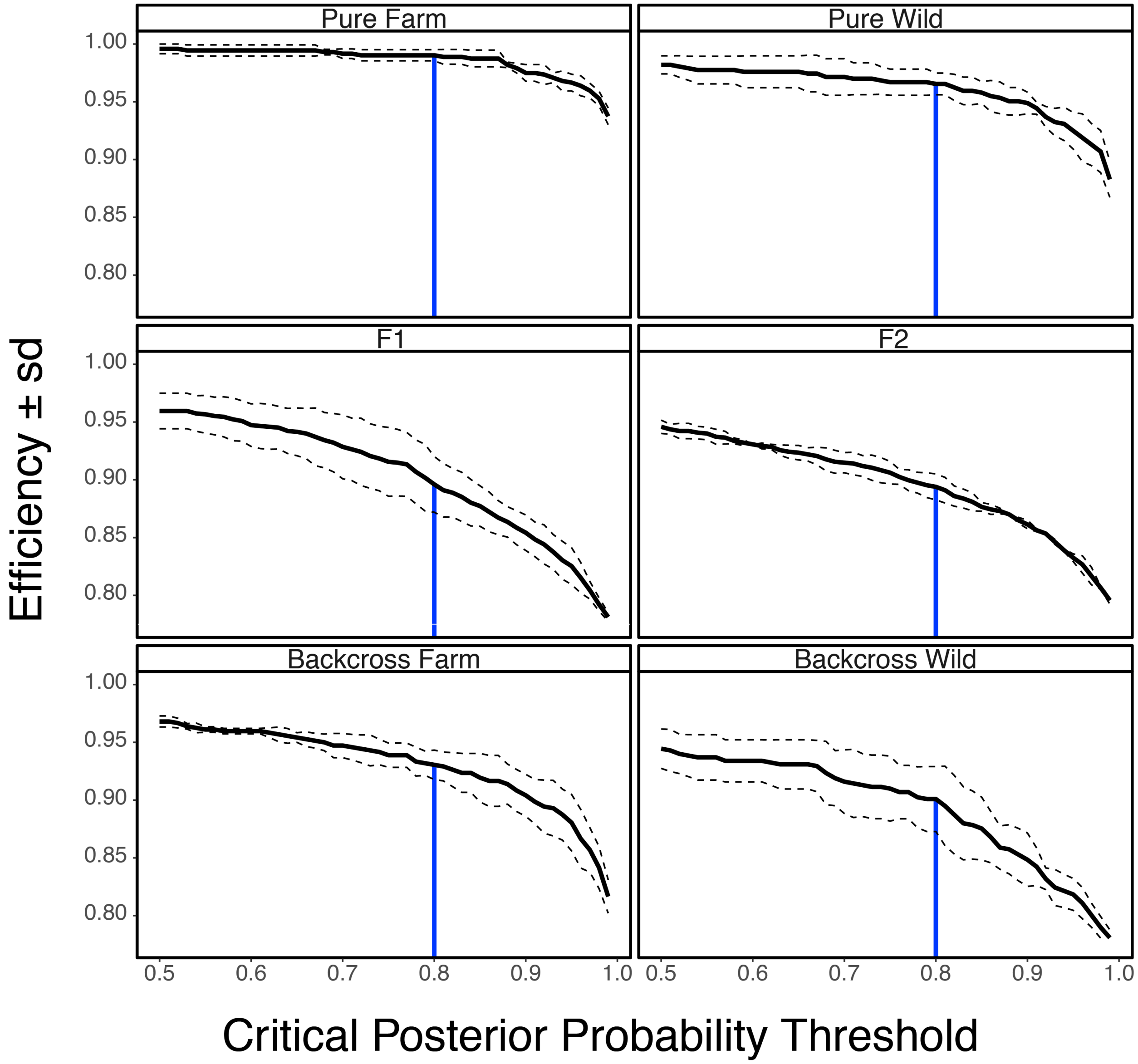

Supplementary Figure 1. Efficiency of detection of each of the genotype frequency classes across a range of critical posterior probability thresholds for the 95 SNP panel used in this study. The black line represents the mean of three replicate analyses of each of three independently simulated datasets and the dotted lines are the standard deviation. The vertical blue line is meant to highlight the critical posterior probability of assignment threshold (> 0.8) used in this study.

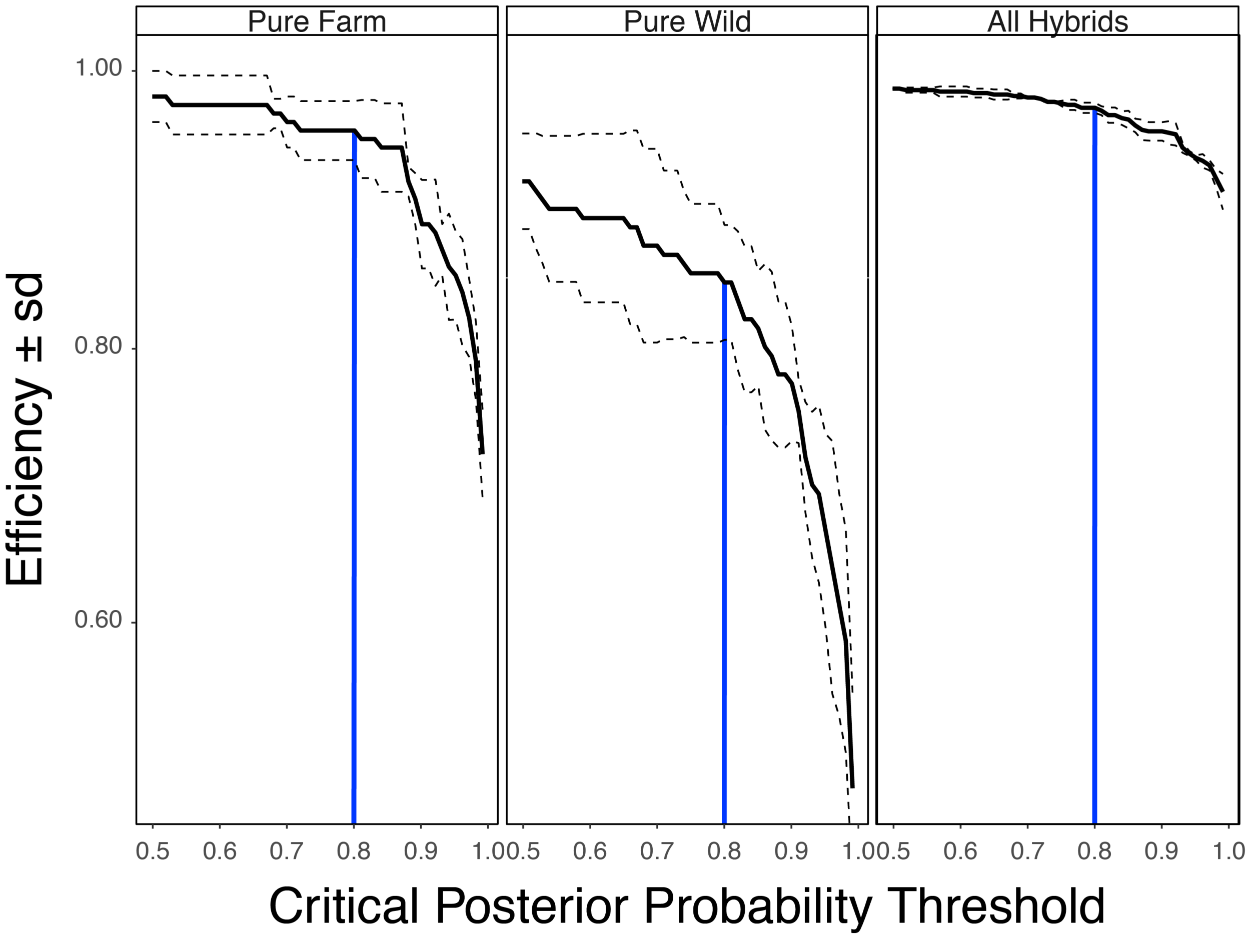

Supplementary Figure 2. Efficiency of detection of the pure genotype frequency classes, and all hybrid genotype frequency classes combined across a range of critical posterior probability thresholds for the 95 SNP panel used in this study. The black line represents the mean of three replicate analyses of each of three independently simulated datasets and the dotted lines are the standard deviation. The vertical blue line is meant to highlight the critical posterior probability of assignment threshold (> 0.8) used in this study.

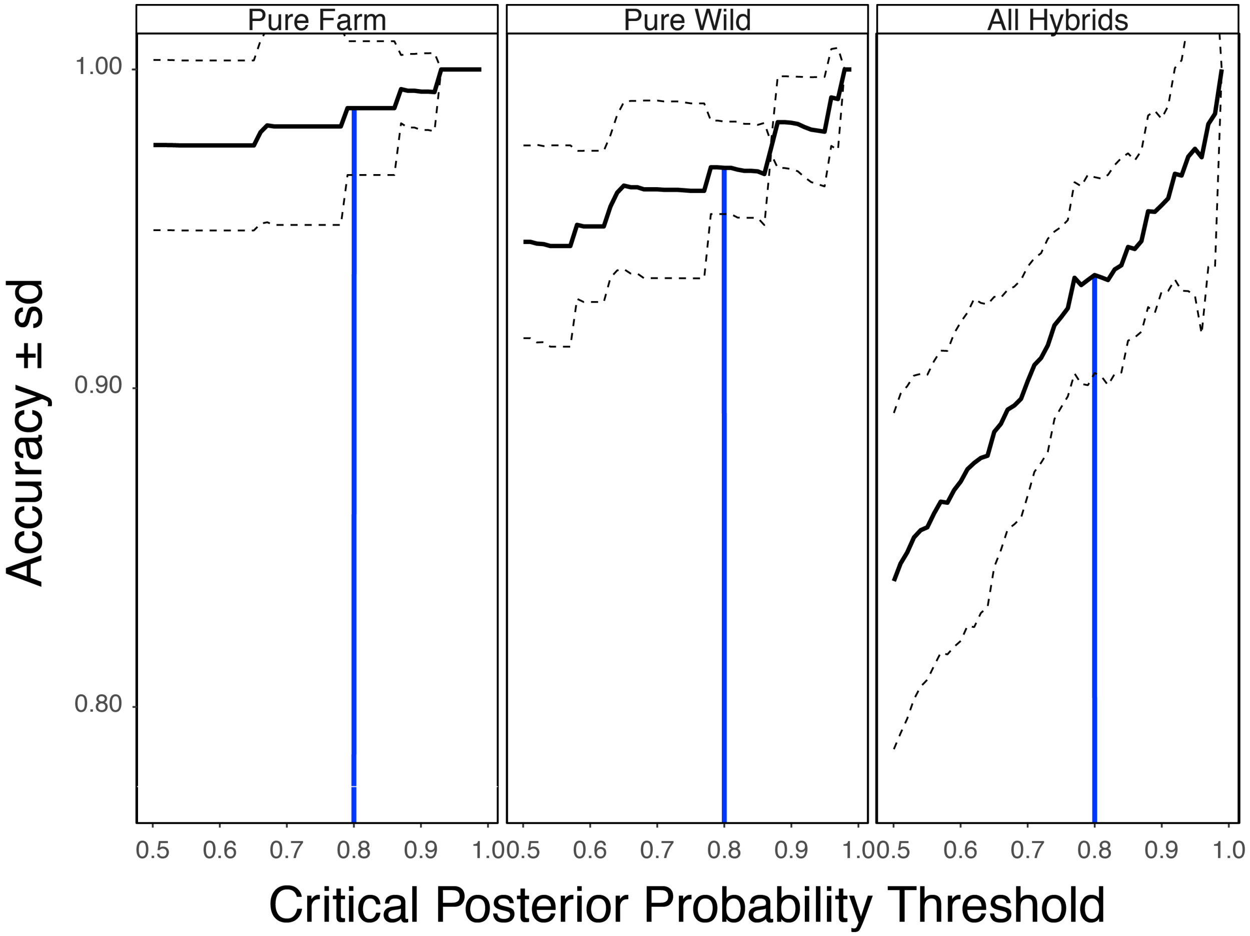

Supplementary Figure 3. Accuracy of detection of the pure genotype frequency classes, and all hybrid genotype frequency classes combined across a range of critical posterior probability thresholds for the 95 SNP panel used in this study. The black line represents the mean of three replicate analyses of each of three independently simulated datasets and the dotted lines are the standard deviation. The vertical blue line is meant to highlight the critical posterior probability of assignment threshold (> 0.8) used in this study.

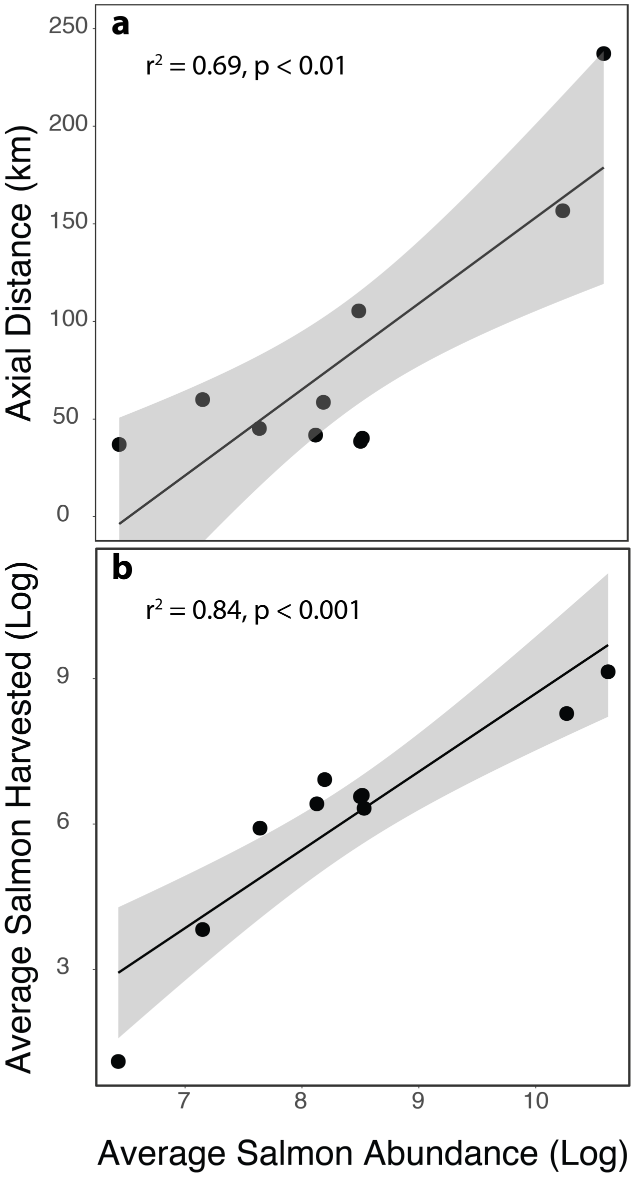

Supplementary Figure 4. a Relationship between river size (axial distance, the distance along a straight line along the longest axis of the river, in km) and the mean number of returning Atlantic salmon detected at Fisheries and Oceans Canada counting fences on the Island of Newfoundland for the period of 2010 to 2014. **b** Relationship between the mean number of salmon caught by recreational anglers and the mean number of Atlantic salmon detected at Fisheries and Oceans Canada counting fences on the Island of Newfoundland for the period of 2010 to 2014. For panel a, the F-statistic was 18.18 on 1, and 8 degrees of freedom, with an intercept of 7.0141 (± 0.3830), and slope 0.0158 (± 0.0037). For panel b, the F-statistic was 40.47 on 1 and 8 degrees of freedom, with an intercept -7.4513 (± 2.153), and a slope 1.632 (± 0.2565).

Supplementary References

1 Porter, T. R., Riche, L. G. & Traverse, G. R. Catalogue of Rivers in Insular Newfoundland. Volume B. 287 (Environment Canada, Fisheries and Marine Service, Resource Development Branch, Newfoundland Region, 1974).

2 DFO. Atlantic Salmon (*Salmo salar*) Stock Status Update in Newfoundland and Labrador for 2014. DFO Canadian Science Advisory Secretariat Science Response 2015/023. (2015).

3 DFO. Stock Assessment of Newfoundland and Labrador Atlantic Salmon – 2016. DFO Can. Sci. Advis. Sec. Sci. Advis. Rep. 2017/035. . (2017).
