# Supplementary material for: Extensive hybridization following a large escape of domesticated Atlantic salmon in the Northwest Atlantic
Source: Commun Biol. 2018 Aug 9;1:108. doi: 10.1038/s42003-018-0112-9 (PMC6123692; doi:10.1038/s42003-018-0112-9)
Supplement: Supplementary file 2 — Description of Additional Supplementary Information [file 42003_2018_112_MOESM2_ESM.docx]

**Description of Additional Supplementary Files**

File Name: Supplementary Data 1

Description: Minor allele frequencies for each SNP in each of the baseline samples. Abbreviations are as in Supplementary Table 5
